# Efficacy of Hydroxyurea in Patients With Sickle Cell Anemia in a Low-Income Country (Côte d'Ivoire)

**DOI:** 10.1155/anem/3576890

**Published:** 2025-03-25

**Authors:** Kouassi Gustave Koffi, Ruth Dieket, Emeraude N'dhatz, Nelly Eloise Abenan, Alexis Dohoma Silué, Ismael Kamara, Boidy Kouakou, Danho Clotaire Nanho

**Affiliations:** ^1^Hematology Teaching Hospital of Yopougon, Abidjan 21 BP 632, Ivory Coast; ^2^University Felix Houphouet-Boigny GPE-Abidjan, Abidjan, Ivory Coast

## Abstract

**Background:** Very few trials of hydroxyurea efficacy and safety have been conducted in sub-Saharan Africa. We aimed to evaluate the efficacy and safety of hydroxyurea and its utility in low-resource settings.

**Methods:** We conducted a prospective comparative trial in patients with SCA. 128 patients were enrolled and divided into two groups. 68 patients were treated with hydroxyurea at a dose of 10–20 mg/kg/day and 62 patients in a control group without hydroxyurea. The endpoints evaluated were feasibility, safety, and benefit (laboratory variables, sickle cell–related events, transfusions).

**Results:** The patients assigned to hydroxyurea treatment had a lower annual rate of crises than the control group (median 2.9 vs. 5.3 crises per year, *p*=0.001), a lower annual rate of hospitalizations (median 2.2 vs. 4.7, *p*=0.002), and a lower annual rate of transfusions (median 1.3 vs. 5.1, *p*=0.001). We observed a significant increase in Hb F from 11.77% to 14.6% (*p*=0.001) in patients treated with hydroxyurea. We also observed a significant increase in the mean Hb level from 7.3 g/dL to 9.2 g/dL in patients treated with hydroxyurea (*p*=0.004). Patients treated with hydroxyurea also have a beneficial effect on WBC and platelet levels by reducing leukocytosis and thrombocytosis. The annual number of infectious complications was significantly lower in the group of patients treated with hydroxyurea.

**Conclusion:** Hydroxyurea has an important clinical benefit by reducing the incidence of vaso-occlusive events, infections, and transfusions, which translates into fewer hospitalizations. The main problem is that it is not accessible to most of our patients who live in poor socioeconomic conditions.

## 1. Introduction

Sickle cell disease is the most common genetic hemoglobin disorder and most commonly affects people of the African descent. Côte d'Ivoire is one of the fifteen most affected countries in the world [1]. Sickle cell disease or sickle cell anemia is an autosomal recessive genetic disorder in which abnormal hemoglobin is inherited. It is a point mutation of hemoglobin resulting from a single mutation in the β-globin gene, leading to an amino acid substitution (Glu⟶Val and βA⟶βS) and formation of the abnormal HbS tetramer. Under deoxygenated conditions, HbS undergoes rapid intracellular polymerization, which damages the erythrocyte membrane and significantly alters both its shape and flexibility as it transports oxygen to the body. These stiff and “sickled” red blood cells have a reduced lifespan and lead to both acute and chronic hemolysis. They also underwent a complex process known as vaso-occlusion, in which sickled erythrocytes and other circulating cells adhere to the vascular endothelium and aggregate to obstruct blood flow, particularly in small blood vessels, causing hypoxia in tissues and vital organs [2].

According to the World Health Organization, the global incidence of sickle hemoglobinopathies is highest in sub-Saharan Africa, with more than 300,000 babies born each year with sickle cell disease, representing approximately 1% of births in the region. Côte d'Ivoire is one of the fifteen most affected countries in the world [3], with the highest sickle cell burden, lack of early diagnosis capacity, and high infant mortality [4]. As recently as the 1980s, the only available disease-modifying therapy for SCA was intermittent red blood cell transfusions, usually reserved for acute, life-threatening complications. In the 1990s, hydroxyurea emerged as a promising pharmacological therapy for SCA, primarily due to its ability to induce HbF, and over the past 30 years has become the primary disease-modifying treatment modality [5]. In several prospective research studies, hydroxyurea has consistently demonstrated improvement in the abnormal laboratory parameters of SCA, while reducing acute clinical complications such as painful vaso-occlusive events and acute chest syndrome, as well as the need for blood transfusions and hospitalization [5, 6]. More recently, hydroxyurea has been associated with improved survival in both adults and children with SCA [6]. In Côte d'Ivoire, the use of hydroxyurea remains limited not only because of the low income of the population, its nonavailability on a large scale, but also because of the initial reluctance of health professionals to use it. We therefore propose to carry out this work whose general objective was to evaluate the effectiveness of hydroxyurea in sickle cell patients in Côte d'Ivoire in terms of its impact on clinical and biological manifestations.

## 2. Materials and Methods

### 2.1. Patients and Inclusion Criteria

The Clinical Hematology Department of the Teaching Hospital of Yopougon (TH-Y) in Abidjan is the referral center for the management of hematological disorders in Cote d'Ivoire. We conducted a prospective comparative study of hydroxyurea in patients with sickle cell anemia. All patients treated for sickle cell anemia at the TH-Y were registered in our database. The study cohort included sickle cell anemia documented by electrophoresis hemoglobin tests, treated with or without hydroxyurea. A total of 128 patients were included in the study, divided into two groups of patients. Group 1 with 66 patients treated with hydroxyurea and Group 2 with 62 patients without hydroxyurea as a control group. The study lasted from April 2019 to May 2023. Demographic, clinical, and laboratory information and data were collected at the time of diagnosis. All patients received routine folic acid, sulfadoxine-pyrimethamine for malaria prophylaxis and penicillin for bacterial prophylaxis. Infants received pneumococcal conjugate vaccine in the first year of life as part of the National Expanded Programme on Immunization.

### 2.2. Treatment With Hydroxyurea

Patients were treated with hydroxyurea according to institutional review board–approved protocols and informed consent was obtained in accordance with the Declaration of Helsinki. 10–20 mg/kg/day of hydroxyurea was prescribed to SCA patients according to disease severity criteria. These criteria were severe or recurrent sickle–related clinical complications such as painful vaso-occlusive crises (VOCs), blood transfusions, hospitalization for sepsis or malaria, and high-risk designation based on prior cerebrovascular accident, acute chest syndrome, or other life-threatening event. Patients starting hydroxyurea were recommended to have a complete blood count after 4–6 weeks of starting hydroxyurea to monitor for myelosuppression whenever possible.

During the study period, hydroxyurea was only available in 500 mg capsules. The number of capsules administered per week depended on the patient's weight. The total dose in milligrams per week was calculated for each patient and divided by 500 to determine the number of capsules administered per week for each patient with split dosing days. Hydroxyurea was administered to young children by opening the capsule and dissolving the contents in liquid or food. Patients were started at a dose of 10 mg/kg/day. The dose was then escalated to 15 mg/kg/day if there were persistent sickle cell crises. If patients experienced events at a dose of 15 mg/kg/day, the dose was escalated to 20 mg/kg/day. The dose was also adjusted on a case-by-case basis to reduce toxicity, monitored by a peripheral blood count to determine a maximum tolerated dose, which was defined as a stable daily dose that caused mild bone marrow suppression without toxic effects.

### 2.3. Clinical Data Collection

All patients were followed up with routine examinations every 3 months and more frequently if clinically indicated. Routine data collected included the occurrence of SCD–related acute clinical crises, blood transfusions, hospitalizations at least 3 per year, and complications. Routine laboratory tests were performed every 6 months, including complete blood count, serum chemistry including renal and liver function, and urinalysis. Patient information was recorded on paper case report forms and stored in individual patient files. Key clinical variables were extracted from the paper forms at the time of analysis and linked to an electronic database of laboratory results.

### 2.4. Statistical Analysis

The following software was used for data entry and analysis: Epi Info 7.2.5.0 for data collection and IBM SPSS software Version 25.0 for statistical analysis. The data were tested for normal distribution. The statistical test used in this study is the Student's *t*-test for paired series. A *p* value of less than 5% (*p* value < 0.05) was considered significant.

### 2.5. Human Ethics and Consent to Participate

Written informed consent to participate in the study was obtained from adult patients or parents of children, and consent was also required for young children in accordance with the Declaration of Helsinki. Approval was granted by the National Research Ethics Committee of Côte d'Ivoire. For young children (10–17 years), informed consent was required in addition to parental or guardian consent.

## 3. Results

### 3.1. Study Population

The study included 128 patients divided into two groups. 68 patients were treated with hydroxyurea and 62 patients were not treated with hydroxyurea. The baseline characteristics of the patients are shown in Table 1. The patients were homogeneously distributed between the two groups. In fact, there were no significant differences between the two groups of patients with regard to age, sex, number of annual VOCs, number of annual hospitalizations, genotype profile, and blood counts in the two groups before the treatment began.

### 3.2. Results by Clinical Manifestations

The ranks of crisis rates were significantly different in the two groups (Table 2), with median annual rates of a vaso-occlusive range of 2.9 crises per year in the hydroxyurea group and 5.3 crises per year in the control group (*p*=0.001). The median annual rate of hospitalization was significantly lower in patients treated with hydroxyurea (2.2 vs. 4.7) than in patients not treated with hydroxyurea (*p*=0.002). The median annual transfusion rate requirement was also significantly lower (*p*=0.001) in the hydroxyurea group (1.3 vs 5.1) per year after treatment.

Regarding the anemic complication profile, we observed 18 cases in all our patients. 8 cases in the hydroxyurea group versus 10 cases in the no hydroxyurea group. These complications included cholelithiasis, splenic sequestration, and a leg ulcer that occurred after 9 months of treatment. However, the incidence of anemic complications was not significantly different between the two groups (*p*=0.48). There was also no difference in the occurrence of ischemic complications between the two groups. Ischemic complications occurred in 18 patients on hydroxyurea and 17 patients off hydroxyurea, mainly retinopathy and osteonecrosis. Two episodes of stroke and priapism occurred in 3 patients who did not have these complications before treatment. However, the annual number of infectious complications was significantly lower in the group of patients on hydroxyurea (16 cases) compared to (30 cases) the group of patients without hydroxyurea (*p*=0.008). Infectious complications included osteomyelitis and pulmonary infection.

### 3.3. Results by Biological Manifestations

In terms of biological characteristics, the whole patients presented homozygous severe form genotypes of sickle cell disease SS with 68% of cases and 32% of genotypes of sickle cell disease S-beta zero thalassemia. In Table 3, we observed a significant increase in the Hb F level from 11.77% to 14.6% (*p*=0.001) in patients treated with hydroxyurea. In patients with the SS genotype, the percentage of Hb F increased by 3.43%, while in patients with the S-beta zero thalassemia genotype, it increased by 1.19%, with a value of *p*=0.0023. We also observed a significant increase in the mean Hb level from 7.3 g/dL to 9.2 g/dL in patients treated with hydroxyurea (*p*=0.004). Patients treated with hydroxyurea also had a beneficial effect on WBC and platelet levels, reducing the leukocytosis and thrombocytosis commonly seen in sickle cell disease.

## 4. Discussion

Hydroxyurea has important clinical benefits, including reducing the frequency of transfusions and the number and intensity of VOCs, which translates into fewer hospitalizations. Indeed, we observed a significant reduction in the number of VOCs. The average annual number of VOCs decreased from 5.2 to 2.9, with a statistically significant difference (*p*=0.001) from the patients treated with hydroxyurea, as previously reported by Mellouli and Bejaoui [7]. These results are also supported by Tshilolo et al. in 2018 [8] who demonstrated that hydroxyurea is a safe daily medication in children with sickle cell disease in Africa, reducing the number of VOCs, infections, transfusions, and deaths. Thomas et al. [9] and Brun, Bourdoulous, and Couraud in 2001 [10] also explained that hydroxyurea helps to stop the inflammatory processes involved in vaso-occlusive events.

The median transfusion requirement rate also decreased significantly in our study (*p*=0.001), from more than 3.4 transfusions per year to 1.3 transfusions at the end of the study. These results are consistent with those of Wang et al. [11] in a pediatric study who reported no transfusions after 12 months of treatment compared to more than three transfusions per year before hydroxyurea.

In the pediatric studies mentioned above, the number of days spent in the hospital decreased significantly during treatment with hydroxyurea. In our study, the median annual hospitalization rate decreased significantly from 3.8 to 2.2. Similar results were reported by Oliveri and Vichinsky [12]. Wang et al. reported that the use of hydroxyurea treatment is associated with substantial savings in medical costs due to its reported benefits over long-term treatment [11]. In fact, these significant reductions in hospital days provide real savings in hospital costs and improve the psychological well-being of patients.

As a result, treatment becomes predominantly outpatient and can be administered at home.

The clinical benefits observed are similarly distributed across the entire population of our study. Indeed, hydroxyurea reduces the number of VOCs and annual transfusions in both patients with homozygous sickle cell disease (SSFA2) and those with heterozygous sickle cell disease (SFA2), without any significant difference.

On the complication profile, there was no significant difference between the two groups in terms of anemic and ischemic complications. 12% of anemic complications were observed, including 3.9% that persisted despite treatment and 0.8% that occurred during treatment with hydroxyurea. These complications included cholelithiasis, splenic sequestration, and a leg ulcer that occurred after 9 months of treatment. Ischemic complications persisted in 8 patients, mainly retinopathy and osteonecrosis. Two episodes of stroke and priapism occurred in 3 patients who did not have these complications before treatment. However, infectious complications were significantly lower in the hydroxyurea groups (16 cases) than in the nonhydroxyurea groups (30 cases), mainly due to osteomyelitis and pulmonary infection.

These observations were also reported in the Western literature. Although hydroxyurea was shown to be effective in reducing VOCs in both adults and children, uncertainties remain regarding its effect on specific organs. It is not known whether hydroxyurea can prevent or correct chronic damage to all organs [13–15] and opinions remain controversial. Some complications with hydroxyurea were reported in a systematic review by Pandey et al. [15].

A study by Mahadeo et al. [16] described the occurrence of aseptic necrosis of the hip during treatment with hydroxyurea. The occurrence of these known complications of sickle cell disease during treatment suggests that hydroxyurea may not prevent all vaso-occlusive phenomena of this disease.

In terms of biological aspects, our patients mainly presented the homozygous severe form (SS) with 68% of cases, followed by subjects with the S-beta zero thalassemia genotype (32%). Studies by Wang et al. [11] and Mellouli et al. [7] also reported similar results, with 96.43% and 57.45% of patients presenting with the severe SS form, respectively. Several arguments suggest that Hb F has a moderating effect on the severity of sickle cell disease. Individuals with sickle cell disease with hereditary persistence of Hb F and patients from certain regions, such as Saudi Arabia and India, with high levels of Hb F have less severe crises. The incidence of specific complications such as hand–foot syndrome, acute chest syndrome, or VOCs is proportional to the decrease in Hb F during the first months of life [17].

The observation that the severity of sickle cell anemia was inversely related to the level of Hb F led to attempts to reactivate the synthesis of Hb F. Hydroxyurea, as the reactivating molecule for the synthesis of the best-tolerated Hb F [17], was investigated in several studies around the world. For example, the study conducted by Mellouli et al. [7] in Tunisia in 2007 showed a significant increase in Hb F levels from 3% to 30% after 6 years and 9 months of treatment with hydroxyurea. Similarly, the study by Tshilolo et al. [8] showed a significant increase in hemoglobin and fetal hemoglobin levels from 11.8% to 13.1%.

In our study, we observed a significant increase in Hb F from 11.70% to 14.6% (*p*=0.001). In SS sickle cell patients, the percentage of Hb F increased by 3.43%, while in SFA2 patients, it increased by 1.19%, with a value of *p*=0.0023. Thus, hydroxyurea contributes to a greater increase in the percentage of Hb F in SSFA2 patients than in SFA2 patients. These results are in agreement with those of the study by Davies et al. [18], who also reported a less significant increase in Hb F in patients with SFA2, but who were better relieved at the pain level.

The beneficial effect of hydroxyurea is not only attributed to the reactivation of Hb F synthesis, as it was at the beginning of its use in sickle cell patients, but is also related to several other processes [5]. Some patients experienced an improvement in their clinical condition without a significant increase in their Hb F levels during treatment. It is suggested that several mechanisms of action of hydroxyurea may benefit sickle cell patients.

We also observed anemia with a significant increase in mean Hb from 7.3 g/dL to 9.2 g/dL (*p*=0.004). Patients also experienced leukocytosis, but there was a statistically significant (*p*=0.001) normalization of the white blood cell count. These results are in agreement with those of Ware et al. [14]. The decrease in all leukocyte subpopulations was more pronounced in neutrophils. The decrease in neutrophil levels could play an important role in CVO, where these cells can be involved in the genesis of vaso-occlusions and inflammatory pathways [19].

Like many authors, we noted the beneficial effect of HU on WBC and platelet levels in terms of a reduction in the leukocytosis and thrombocytosis usually observed in sickle cell disease. This reduction is beneficial for blood rheology. However, the risk of cytopenia justifies close and regular monitoring of the blood counts of patients receiving HU.

It is important to emphasize the clinical benefits of high adherence to hydroxyurea, especially in young patients with sickle cell disease major. Recent studies in the United States showed that hydroxyurea is well tolerated in young children, leading some to recommend its use from the first year of life. However, the main problem associated with hydroxyurea is the variability of its effectiveness from one patient to another. In fact, clinical improvement is not always directly related to the increase in Hb F levels. This suggests that although the reactivation of Hb F synthesis plays an important role in the action of hydroxyurea, the improvement in the general condition is also linked to other factors. This “paradox” can be explained by other beneficial effects of HU, such as the reduction of leukocytes, platelets, and reticulocytes, and the increase in Hb and MCV [13, 17].

Over the years, other mechanisms of action have been suggested: reduction of excessive adhesion of sickle cell reticulocytes to the endothelium, modulation of inflammatory processes, cellular effects (reduction of dense cells), and induction of NO [10].

### 4.1. Safety

Our patients tolerated a mean HU dose of 17.1 ± 3.4 mg/kg/day. This suggests that the maximum tolerated dose is not necessary to achieve a therapeutic effect. The mean dose of HU used in the study by Tshilolo et al. in 606 children was 17.5 ± 1.8 mg/kg/day [8]. Therefore, the dose must be adjusted on a case-by-case basis to reduce toxicity. The initial dose is 10–15 mg/kg/day in one dose up to a maximum of 30 mg/kg/day [20].

We observed 2 cases of rash and 2 cases of hyperpigmentation. The most common short-term toxicity is mild and reversible cytopenias. In general, toxic events occurred in 5.1% of participants, which is below the protocol safety threshold during treatment. [12]. Although all cell lines may be affected, the most common hematological toxicities include mild to moderate neutropenia, followed by reticulocytopenia and thrombocytopenia [12, 15]. This risk of cytopenia justifies regular hematological monitoring in patients receiving HU. No mortality or carcinogenic effects were observed in our study or in previous studies [21]. Ware et al. [14] also demonstrated the safety of HU in infants. However, the absence of malignant processes in our study does not guarantee the reliability of the long-term safety of this treatment.

## 5. Conclusion

Our study confirms the important clinical benefits of hydroxyurea, including reducing the frequency of transfusions and the number and intensity of VOCs, which translates into fewer hospitalizations. Hydroxyurea treatment was feasible and safe for children with sickle cell anemia in low-income countries such as sub-Saharan Africa. The benefits observed are a reason for the prescription. However, hydroxyurea does not appear to completely prevent or correct all the chronic complications of sickle cell disease. Its toxicity is modest in the short and medium term but uncertain in the long term, which justifies regular clinical and biological monitoring and the need for long-term studies.

## Figures and Tables

**Table 1:** Characteristics of the patients at baseline according to the treatment group.

Characteristics	Hydroxyurea *n* = 66	No hydroxyurea *n* = 62
Age. Mean (years) ± SD	10.5 ± 7	11.2 ± 6
Gender *n*		
Male	41 (62%)	42 (68%)
Female	25 (38%)	20 (32%)
No. VOC/years		
3–5	27	27
6–8	24	23
> 8	15	12
Mean value ± SD	5.2 ± 1.1	5.7 ± 1.2
No. hospitalization/years		
3–5	35	33
6–8	29	26
> 8	2	3
Mean value ± SD	3.8 ± 1.4	3.5 ± 1.8
Transfusion/years		
3–5	50	48
≥ 6	16	14
Mean value ± SD	3.4 ± 1.2	3.7 ± 1.3
Hemoglobin g/dL (mean ± SD)	7.3 ± 1.05	7.6 ± 1.1
Hématocrit % (mean ± SD)	22.3 ± 3.7	23.1 ± 2.6
Leukocyte count (G/L) (mean ± SD)	12.2 ± 4.1	11.3 ± 5.2
Platelet count (G/L) (mean ± SD)	358.665 ± 142.588	311.435 ± 123.167
Electrophoretic profile		
SS	45 (68%)	43 (69%)
SFA2	21 (32%)	19 (31%)
% Hb F (mean ± SD)	11.7 ± 7.7	12.2 ± 2
% Hb S (mean ± SD)	86 ± 7.8	82 ± 4.5

**Table 2:** Impact of hydroxyurea on clinical manifestations.

Parameters	Hydroxyurea *n* = 66	No hydroxyurea *n* = 62	*p* value	Significant
VOC rate/years (median ± SD)	2.9 ± 0.7	5.3 ± 1.1	0.001	S
Hospitalization rate/years (median ± SD)	2.2 ± 0.5	4.7 ± 1.4	0.002	S
Transfusion rate/years (median ± SD)	1.3 ± 0.7	5.1 ± 1.5	0.001	S
Anemic complications (*n*.%)	8 (12%)	10 (16%)	0.48	NS
Ischemic complications (*n*.%)	18 (27%)	17 (27%)	0.74	NS
Infectious complications (*n*.%)	16 (24%)	30 (48%)	0.008	S

*Note:* The annual median hospitalization rate was significantly lower in the group of hydroxyurea than in the group without hydroxyurea (2.2 ± 0.5 vs 4.7 ± 1.4). This was also found for the annual median transfusion rate (1.3 ± 0.7 vs 5.1 ± 1.5) compared in the two group.

**Table 3:** Influence of hydroxyurea on biological parameters.

**Parameters**	**Patient with hydroxyurea**	**Patient without hydroxyurea**	**p** **value**	**Significant**

Hemoglobin g/dL (mean ± SD)	9.2 ± 1.1	7.2 ± 1.1	0.004	S
Hematocrit % (mean ± SD)	28.3 ± 18.7	22.3 ± 3.7	0.001	S
Leukocyte count (G/L) (mean ± SD)	9.5 ± 4.8	12.6 ± 3.1	0.001	S
Platelet count (G/L) (mean ± SD)	316.4 ± 130.1	358.6 ± 142.6	0.005	S
% Hb F	14.6 ± 7.2	12 ± 7.8	0.001	S
% Hb S	84.3 ± 11.9	86.2 ± 7.9	0.001	S

## Data Availability

The data used to support the findings of this study are available from the corresponding author upon request. The data are not publicly available due to privacy or ethical restrictions.

## References

[1] Ware R. E., de Montalembert M., Tshilolo L., Abboud M. R. (2017). Sickle Cell Disease. *The Lancet*.

[2] Bunn H. F. (1997). Pathogenesis and Treatment of Sickle Cell Disease. *New England Journal of Medicine*.

[3] https://www.afro.who.int/publications/who-sickle-package-intervention-sickle-cell-disease-management.

[4] Piel F. B., Patil A. P., Howes R. E. (2013). Global Epidemiology of Sickle Haemoglobin in Neonates: a Contemporary Geostatistical Model-Based Map and Population Estimates. *The Lancet*.

[5] Charache S., Terrin M. L., Moore R. D. (1995). Effect of Hydroxyurea on the Frequency of Painful Crises in Sickle Cell Anemia. *New England Journal of Medicine*.

[6] Steinberg M. H., Barton F., Castro O. (2003). Effect of Hydroxyurea on Mortality and Morbidity in Adult Sickle Cell Anemia: Risks and Benefits up to 9 Years of Treatment. *JAMA*.

[7] Mellouli F., Bejaoui M. (2008). The Use of Hydroxyurea in Severe Forms of Sickle Cell Disease: A Study of 47 Case in Tunisian Pediatric Cases. *Archives de Pediatrie: organe officiel de la Societe francaise de pediatrie*.

[8] Tshilolo L., Tomlinson G., Williams T. N. (2018). Hydroxyurea for Children with Sickle Cell Anemia in Sub-saharan Africa. *New England Journal of Medicine*.

[9] Thomas R., Dulman R., Lewis A., Notarangelo B., Yang E. (2019). Prospective Longitudinal Follow-Up of Children with Sickle Cell Disease Treated with Hydroxyurea since Infancy. *Pediatric Blood and Cancer*.

[10] Brun M., Bourdoulous S., Couraud P. O. Effect of Hydroxyurea on Endothelial Cells in Culture.

[11] Wang W. C., Oyeku S. O., Luo Z. (2013). Hydroxyurea Is Associated with Lower Costs of Care of Young Children with Sickle Cell Anemia. *Pediatrics*.

[12] Olivieri N. F., Vichinsky E. P. (1998). Hydroxyurea in Children with Sickle Cell Disease: Impact on Splenic Function and Compliance with Therapy. *Journal of Pediatric Hematology*.

[13] Charache S. (1994). Experimental Therapy for Sickle Cell Disease: Use of Hydroxyurea. *American Journal of Pediatric Hematology Oncology*.

[14] Ware R., Steinberg M. H., Kinney T. R. (1995). Hydroxyurea: An Alternative to Transfusion Therapy for Stroke in Sickle Cell Anemia. *American Journal of Hematology*.

[15] Pandey A., Kaur H., Borah S. (2022). A Systematic Review on Hydroxyurea Therapy for Sickle Cell Disease in India. *Indian Journal of Medical Research*.

[16] Mahadeo K. M., Oyeku S., Moody K. (2008). Hydroxyurea Use Is Associated with Avascular Necrosis of the Femoral Head Among Children with Sickle Cell Disease. *Blood*.

[17] Charache S. (1990). Fetal Hemoglobin, Sickling, and Sickle Cell Disease. *Advances in Pediatrics*.

[18] Davies S. C., Gilmore A. (2003). The Role of Hydroxyurea in the Management of Sickle Cell Disease. *Blood Reviews*.

[19] Allali S., Galactéros F., Oevermann L. (2024). Hydroxyurea Is Associated with Later Onset of Acute Splenic Sequestration Crisis in Sickle Cell Disease: Lessons from the European Sickle Cell Disease Cohort-Hydroxyurea (ESCORT-HU) Study. *American Journal of Hematology*.

[20] Ogu U. O., Mukhopadhyay A., Patel K. (2024). Hydroxyurea at Escalated Dose versus Fixed Low-Dose Hydroxyurea in Adults with Sickle Cell Disease. *European Journal of Haematology*.

[21] De Montalembert M., Davies S. C. (2001). Is Hydroxyurea Leukemogenic in Children with Sickle Cell Disease Blood. *Blood*.

[22] Koffi K. G., Dieket R., N’dhatz E. Efficacy of Hydroxyurea in Patients with Sickle Cell Anaemia in a Low-Income Country (Côte d’Ivoire).

